# The pituri story: a review of the historical literature surrounding traditional Australian Aboriginal use of nicotine in Central Australia

**DOI:** 10.1186/1746-4269-6-26

**Published:** 2010-09-12

**Authors:** Angela Ratsch, Kathryn J Steadman, Fiona Bogossian

**Affiliations:** 1 School of Nursing and Midwifery, The University of Queensland, Herston Campus, Brisbane, Australia; 2 School of Pharmacy, The University of Queensland, St Lucia Campus, Brisbane, Australia

## Abstract

The harmful outcomes of nicotine self administration have been the focus of sustained global health education campaigns that have targeted tobacco smoking and to a lesser extent, smokeless tobacco use. 'Smokeless tobacco' infers that the nicotine is not burnt, and administration can be through a range of methods including chewing.

The chewing of wild tobacco plants (*Nicotiana *spp.) is practiced across a broad inland area of Central Australia by traditional Aboriginal groups. Collectively these plants are known by a variety of names - one common name being 'pituri'. This is the first paper to examine the historical literature and consider the linkage between pituri use and health outcomes. Using a narrative approach, this paper reviews the literature generated since 1770 surrounding the term pituri and the behaviours associated with its use. The review examines the scientific literature, as well as the diaries and journals of nineteenth century explorers, expedition notes, and early Australian novels to expound the scientific evidence and broaden the sense of understanding related to pituri, particularly the behavioural elements. The evaluation considers the complexities of ethnobotany pertaining to language and distance and the ethnopharmacology of indigenous plant usage. The review compares the use of burnt and smokeless tobacco to pituri and establishes the foundation for research into the clinical significance and health outcomes of pituri use. Additionally, this review provides contemporary information for clinicians providing care for patients who chew pituri.

## Review

### The pituri story: a review of the historical literature surrounding traditional Australian Aboriginal use of nicotine in Central Australia

Nicotine is the primary pharmacologically active constituent of the tobacco plant, the absorption of which poses significant risks to health including increased platelet aggregation, increased cardiac rate and contractility, stimulation of the adrenal cortex and medulla, and increased release of hypothalamic and pituitary hormones [1-3]. Expedited by the work of Doll and Hill [1] the dominant focus for public health research and consequently health education campaigns, has been on the effects of inhaled burnt tobacco. Nicotine administration by other practices, collectively referred to as smokeless tobacco use [2], includes chewing, dermal pasting and nasal snuff and is relatively uncommon in Western cultures. However, in the traditional indigenous cultures of continental Asia, Indonesia, Papua New Guinea, South America, Africa and Australia, the preferred means of nicotine delivery is often via smokeless routes [3]. The 1986 sentinel report *The Health Consequences of using Smokeless Tobacco *[2] detailed the health outcomes of smokeless tobacco use. The Report, whilst considering a range of smokeless tobacco products and the effects of smokeless tobacco use on the general population, did not examine the use of the wild tobacco plants in Australia.

In Central Australia, Aboriginal people habitually chew wild tobacco plants (*Nicotiana *spp.) for its pharmacologically active nicotine content. These wild tobacco plants are now colloquially and collectively known by a variety of names - one common name being *pituri *[4]. This paper considers the historical literature in order to provide a conceptual foundation for Australian research into the potential health effects of the mastication and transdermal use of pituri.

### The recorded history

It is in Joseph Banks' notes from the 26^th ^August 1770 [5] that the first documentation of Aboriginal chewing is found:

We observd that some tho but few held constantly in their mouths the leaves of an herb which they chewd as a European does tobacca or an East Indian Betele. What sort of plant it was we had not an opportunity of learning as we never saw any thing but the chaws which they took from their mouths to shew us; it might be of the Betele kind and so far as we could judge from the fragments was so, but whatever it was it was usd without any addition and seemd to have no kind of effect upon either the teeth or lips of those who usd it.

**Figure 1:**
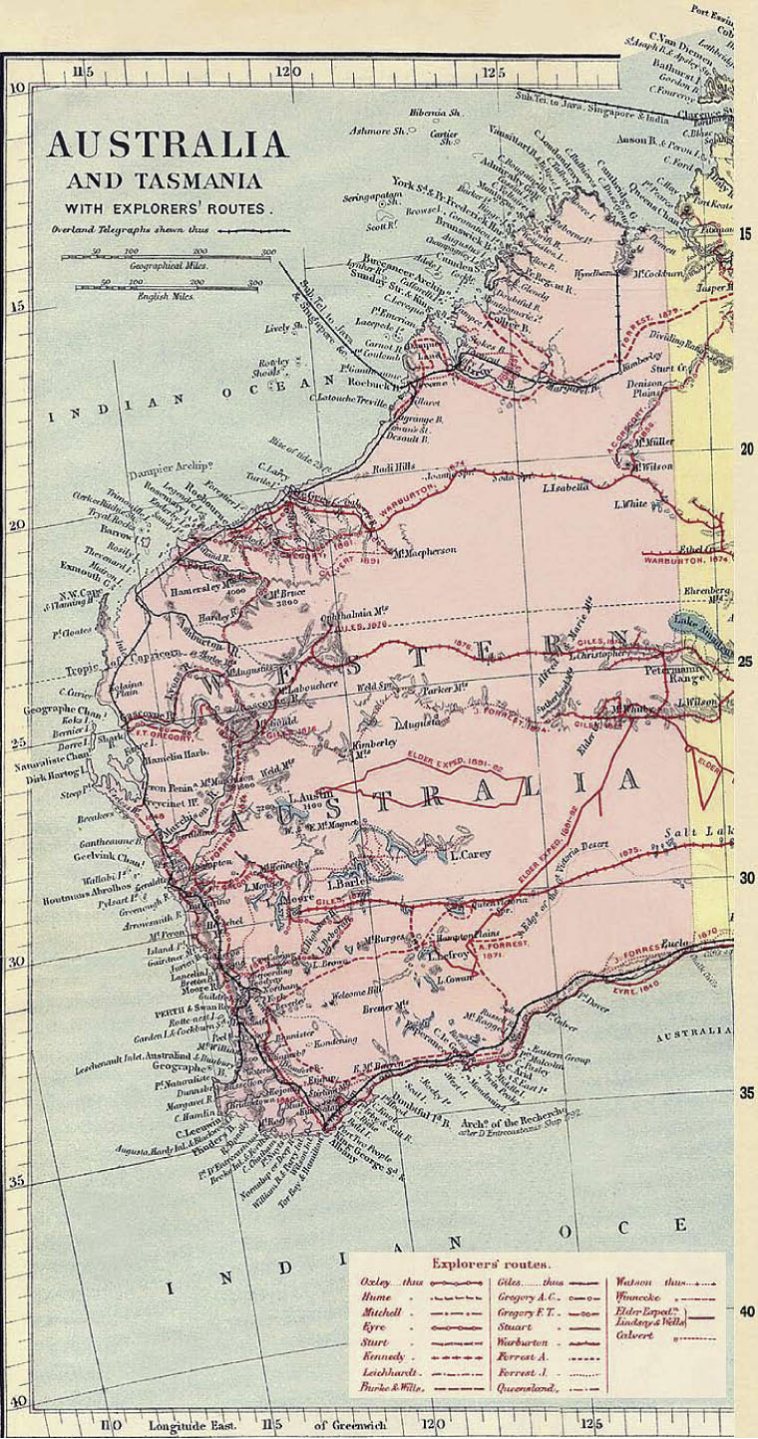
**Part 1 - Map of Nineteenth Century European exploration of Australia (with permission) **[71].

**Figure 2:**
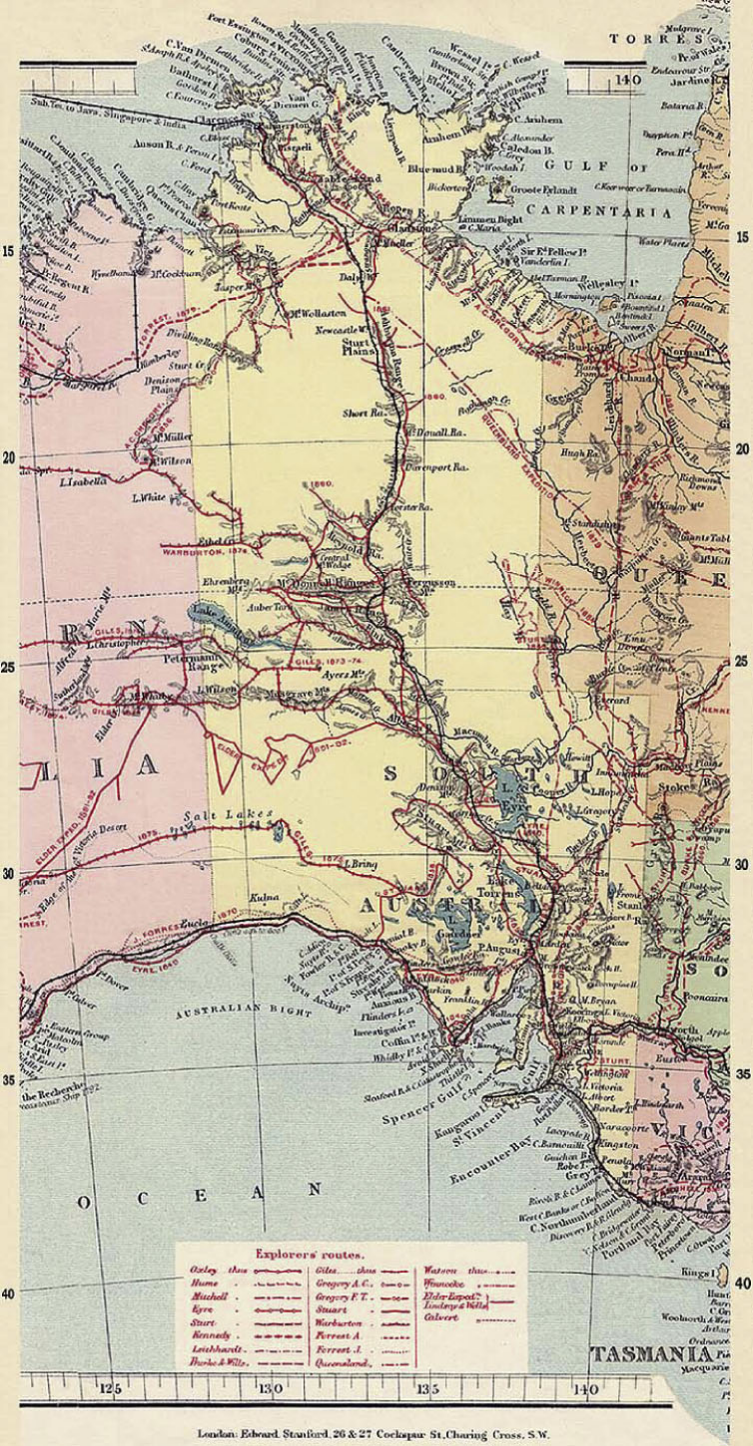
**Part 2 - Map of Nineteenth Century European exploration of Australia (with permission) **[71].

**Figure 3:**
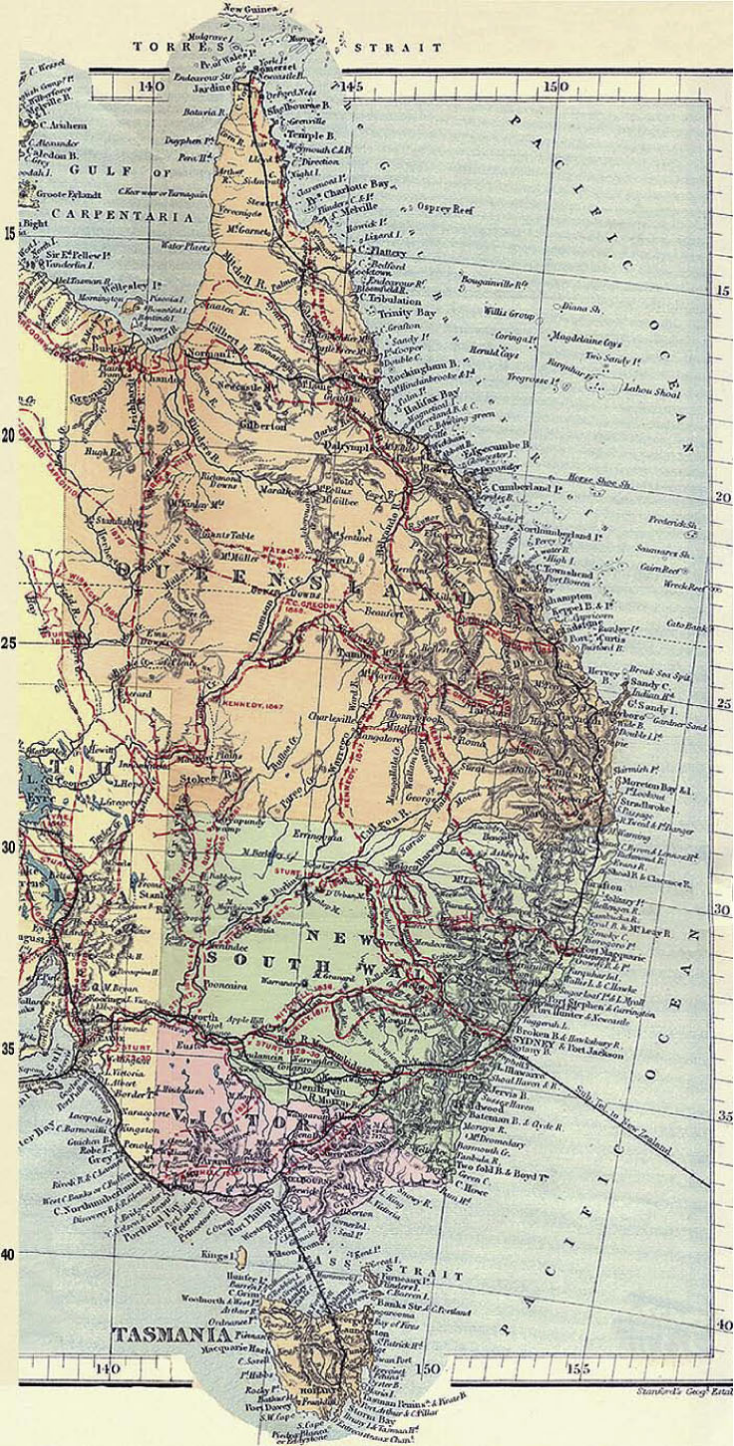
**Part 3 - Map of Nineteenth Century European exploration of Australia (with permission) **[71].

Edmund Kennedy's 1847 diary [6] of his journey west of the Barcoo River (Figures 1, 2 and 3 ) records Aboriginal people chewing 'a leaf similar in taste and smell to Tobacco' and 'it is of course in a green state but it tasted strong and hot'.

Little scientific attention seems to have been directed to these notations until, on the 15^th ^September 1861, the surviving member of the Australian Burke and Wills expedition - Private John King - was discovered by a rescue party lead by Alfred Howitt at Cooper's Creek in Central Australia [7]. Though bedraggled and starved, King had retained the diary of his deceased fellow explorer, William Wills. The diary recounted how, at Camp No. 9 on the 7^th ^of May 1861, when the Burke and Wills party were facing punishing conditions, a group of Aboriginal people came to their assistance. The Aboriginal group fed them fish, bread and a 'stuff they call **bedgery **or **pedgery**; it has a highly intoxicating effect when chewed even in small quantities. It appears to be the dried stems and leaves of some shrub' [8]. This brief record immediately drew the attention of the scientific community. Hicks in 1963 [9] describes the phenomena surrounding the search for the botanical nature of this chewed substance as the 'veritable nineteenth-century scientific romance, and one, moreover, that dealt with an unsolved mystery'. The chewing of the Aboriginal substance was recorded as inducing a broad range of effects - enabling old men to act as seers [10], allowing Aboriginal people to walk hundreds of kilometres without food or water [11], and to 'excite their courage in warfare' [12]. The claim that Aboriginal people 'will usually give anything they possess for it' [13] implied either a level of habituation or addiction.

### The pituri trail

In retrospect, the search for pedgery or *pituri*, by the European explorers and scientists embodies the scientific difficulties encountered in the quest to survey, sample and describe an unknown, sparsely inhabited country. The quasi-ethnographers became confounded in seeking to understand the names and the usage of flora from inhabitants who spoke an extensive range of languages and dialects (but not English) and who employed a diverse range of sign languages across Australia to describe the same entity. The explorers would be tested as they attempted to preserve specimens in an identifiable state and condition for later analysis whilst navigating through deserts and rivers. Furthermore, the scientists were challenged with the complexities of interpreting botanical samples that may have, as described by Liversidge in 1880 [14], endured a journey from the Barcoo in Western Queensland, 'some months in transit, as it had to be carried down on camels to Port Augusta [and then] the sea journey from Port Augusta to Sydney'. Peterson [15] points out the analysis were often completed inaccurately as:

most authors who have written about Aboriginal foods were not botanists... consequently, while the genus is usually correct, the species name is frequently wrong: there is simple misidentification in the field; there is reclassification and change in nomenclature since the author published; and there are the confusions introduced by Europeans using Aboriginal names, the best example of which is the history of the identification of Aboriginal chewing tobaccos [pituri].

It would be nearly 75 years before the exact nature of the substance(s) being chewed by Aboriginal people was known.

### The language of pituri

The fundamental tenet in appraising the historical information surrounding pituri is to recognise that the literature has been formulated from a European perspective. Equivalently, this discourse is from within and comes through a textually mediated European paradigm. The Aboriginal culture, whilst having an extraordinary oral history, is not supported with an extensive written record. Thus the Europeans, without command of the hundreds of languages and two to three times as many dialects, relied upon Aboriginal interpreters for accurate information about all aspects of Aboriginal life including the use of pituri.

A search of the literature around the word pituri highlights the difficulties related to pronunciation. Roth [16] pointed out that the letters *p *and *b *as well as *d *and *t *are interchangeable in the Aboriginal dialects in the Central Australia regions where *pedgery *grows. Compounding the linguistic challenges is that the European writers of the day took extensive phonetic license with the spelling of pedgery (Table 1), thus complicating a search of the literature on the subject. The founder of Australian pharmacology, Joseph Bancroft [17] extracted a potent poison he referred to as 'Pituri' from a sample of supposed pedgery obtained near Bedourie. Bancroft appears to be the first to use *pituri *as the specific spelling. In current literature, this nomenclature has remained.

**Table 1:** Phonetic spelling of pituri in the literature since 1861

Spelling	Reference
bedgery	[8]
betcheri	[72]
boodjerrie	[72]
boodjerre	[72]
budgerie	[72]
budgeri	[72]
bedgeree	[42]
bidgeree	[42]
pecherie	[73]
pecheringa	[73]
pedgery	[8]
petchere	[11]
petcherie	[21]
peturr	[74]
petury	[11]
picherie	[14]
pidgery	[75]
pitchera	[76]
pitcheri	[21]
pitcherie	[77]
pitcherrie	[78]
pitcherry	[79]
pitchery	[8]
pitchiri	[42]
pitchiry	[14]
pitchuri	[80]
pitchurie	[10]
piteri	[18]
pitjiri	[42]
pitjuri	[42]
pituri	[17]
piturie	[42]
piturr	[42]
piturrba	[42]
pitury	[12]
puljantu	[42]

In addition to the spelling and pronunciation of pituri, there has also been confusion related to the exact nature of pituri. This was not helped by Wills' observation [8] on the 3^rd ^June 1861 when on the banks of the Cooper he notes '...I could see smoke, and was shortly afterwards set at my ease by hearing a cooey from Pitchery, who stood on the opposite bank and directed me round the lower end of the waterhole...' and then later on the same date '...when Pitchery, allowing me a short time to recover myself, fetched a large bowl of the raw nardoo...'. Furthermore, Aiston [18] claimed that the name pitcheri is equivalent to a European surname and that it belonged to every boy of the pitcheri moora. For example, the oldest man was Pitcheri Pinnaru and that others were 'called from any distinguishing feature' as in the instance quoted by the explorer Howitt [18] Pitcheri Coona Milkie - meaning one-eyed Pitcheri. No further notations of pedgery, bedgery or pitchery are found in Wills' diary and whilst King (the sole survivor of the expedition) made no mention of the substance in his own *Narrative *[19], Dr Murray, a member of the Howitt rescue party which discovered King, recalled King's use of pituri in his 1879 letter to the Lancet [20].

It proved difficult for the Europeans to comprehend the issues around the ethnobotany and precise information about the localities and preparation of pituri, and, coupled with the linguistic and geographic difficulties in identifying pituri, scientists at this point made assumptions based on two misleading premises. Firstly, that any substance being chewed across Australia was the fabled pituri and, compounding the first premise, that the substance would be chemically identical across Australia.

### Ethnobotanical confusion: *Duboisia *or *Nicotiana*?

Robert Brown (a journeyman with Matthew Flinders) whilst on the 1802-1805 expedition, collected and named a genus of plant *Duboisia *after the French botanist Dubois [21] and the specific plant *Duboisia myoporoides *in his 1810 Prodomus [22]. Dr Beckler, the medical officer/botanist on the Burke and Wills expedition collected samples of different plants from the Cooper's Creek area, one of which the Baron Ferdinand von Mueller in 1861 named *Anthocercis hopwoodii *[23] in honour of Mr. Hopwood of Echuca, who was a sponsor of the Victorian expedition sent in search of Burke and Wills [24]. In 1872, Giles brought back samples of this same plant (which contained the flowers and seeds) from Mt Liebig, north of Alice Springs, which von Muller examined and was able to place the species in the genus *Duboisia*, thus the plant was renamed *Duboisia hopwoodii *[12]. At the same time Joseph Bancroft, a clinical physician, microbiologist, and ethnobotanist in Brisbane had obtained sufficient 'pituri' from Inspector Gilmour near Eyre's Creek and undertook the first detailed pharmacological investigation of a pituri specimen. Bancroft [17] described how minute amounts given as infusions were toxic to frogs, rats, cats and dogs with death following respiratory arrest:

When a quarter to half a drop of the extract diluted with water has been injected under the skin of a rat, the following symptoms are observed:- In less than one minute, the animal becomes very excitable, and jumps and starts with the slightest provocation...shortly, irregular muscular motions occur, passing rapidly into a general convulsion. The animal opens its mouth as if to breathe, but no regular respiratory act follows. Opisthotonos is well marked in some cases. After a few seconds of quiet from muscular effort...a gasp of breath follows which is generally a sign that the poison will not prove fatal. This is succeeded by others, and very shortly rapid respiration takes place...the animal now gradually regains consciousness. In cats and dogs... vomiting of a violent kind occurs.

In 1877 following a lengthy wait for further pituri specimens to come from the inland, Bancroft received a supply collected by the explorer William Hodgkinson during his north-west expedition of Queensland [25]. [It should be noted that the sample was obtained from a live plant and was Hodgkinson's first sighting of the (supposed) plant in a four-month expedition and was gathered without Aboriginal verification that this was the fabled pituri plant]. Hodgkinson's empirical evidence in a letter to Bancroft [21] added further to the intrigue surrounding the nature of pituri:

...your remarks as to the toxicological properties of petcherie must I confess astonish me. Sixteen years ago, when with Burke and Wills expedition, subsequently with Mr McKinlay and recently in the north west expedition, I used petcherie habitually when procurable in default of tobacco and have often chewed it both in its raw and prepared state.

Ferdinand von Mueller [12] examined the Hodgkinson/Bancroft specimens and identified that pituri was in fact the broken leaves and twigs of *D. hopwoodii *which Bancroft [26] described as a shrub or small tree with smooth, very narrow leaves up to 10 cm long, bell-shaped flowers with five petals and three reddish lines running down the throat of the flower.

Bancroft took his pituri to Europe; to Professor Fraser in Edinburgh, Dr Ringer in England and the Parisian chemist Petit. Ringer passed it onto Gerrard, who isolated a volatile alkaloid, and named it 'piturine'. Ringer and Murrell [27] in 1878 had determined that whilst piturine manifested many of the properties of atropine, it still differed from atropine, and in further work in 1879 they demonstrated piturine to be an antidote to the action of muscarine and pilocarpine. Ringer and Murrell considered that pituri 'therefore is more closely allied to tobacco' [28]. Von Muller in 1879 [14] disputed this and said that the 'piturine is in some respects allied to nicotine, but is more closely akin to the duboisine of *D*. *myoporoides*'. (The other notable plant in the genus is *D. myoporoides*. It was discovered to contain an atropine-like alkaloid - sometimes hyoscine, sometimes hyoscyamine and sometimes both. Hyoscyamine in the older tissues, scopolamine in the younger leaves [23]. Subsequently these findings led to the establishment of *D. myoporoides *plantations in Queensland that today still supply the bulk of the world's raw scopolamine [24]).

Meanwhile following experimentation, Petit in 1879 declared that piturine was in fact nicotine [20]. The contention that pituri contained nicotine startled Bancroft who had already compared piturine to nicotine, and found 'the pituri extract is...very much stronger than tobacco extract' [20]. In 1880 at Sydney, Liversidge verified Bancroft and von Muller findings and argued that Petit's conclusion was made on insufficient evidence and that pituri differed in some of its reactions from nicotine [14]. Ten years later in 1890 and with the debate still unresolved, Langley and Dickinson [29] in England obtained a specimen from Liversidge and asserted to the Antipodeans that 'there was no obvious difference between its action and that of nicotin[e]'. The scientific community were still enthralled with the enigma of pituri's exact pharmacological basis. Another ten years of experimentation later, and fifty years after the Burke and Wills expedition, Rothera in 1911 [30], insisted that pituri was indeed nicotine, and he used the term 'catalepsy' to describe the loss of power following injection of piturine into frogs.

Confirmation that Aboriginal people chewed plant substances in a manner similar to European tobacco chewing had been coming in across the broad expanse of Central Australia. Howitt in 1861-1877 reported chewing from northern New South Wales and western and southern Queensland [31,32], Smyth [33] from the Cooper's Creek area in 1876 and Helms [34] from the Elder Exploring Expedition of northwest South Australia and the Great Western Desert of Western Australia (see Figures 1, 2 and 3). Roth [16] gave extensive supporting reports from western Queensland and Carnegie [35] from central Western Australia, with Spencer and Gillen [36] providing further evidence from the western and central Northern Territory area. Interestingly, Bedford [37] recounts the practice of chewing across a wide area in western Queensland but notes in relation to the actual pituri plant 'on Pituri Creek none whatever grows, being only another instance of a misnomer so noticeable in the names of Queensland creeks'.

From Western Australia came an account that the smoke from burning pituri leaves was used by Aboriginal people as 'an anaesthetic for such...operations as they performed' [38]. Importantly, information that Aboriginal people also chewed wild tobacco plants began to emerge. On the Elder Expedition of 1891, Helms [34] observed that:

to find that the natives...use tobacco was a surprise to me. It stuck me as peculiar when I noticed their lips and the corners of their mouth being colored with a yellowish-green rim, and attributed it at once to some peculiar food they might have been eating, but later on I discovered that it's true cause was the sucking of a roll of native tobacco...Whilst these tribes have discovered the stimulating properties of *Nicotiana suaveolens*, they do not seem to know the more powerful narcotic of 'pituri' *Duboisia Hopwoodii*, which also occurs in many places throughout the same regions.

Heightening the interest in the pharmacological compounds of pituri, particularly Bancroft's findings of toxic substances, were reports coming in that Aboriginal people also used *D. hopwoodii *as a poison and that cattle and sheep which ate it died [38]. Hicks and Le Messurier [39] claimed that 'it is well-known [that camels] succumb if they eat only one mouthful of the bush torn off during a journey.' Kempe in 1882 [40] observed of *D. hopwoodii *that 'the leaves of this shrub are used by the natives to poison emus' around the Hermannsburg area of Central Australia. This observation was substantiated by Schulze [41] on his journey through the Finke River areas, and Spencer and Gillen's seminal work *The Native Tribes of Central Australia 1899 *[36] describes how the:

leaves of the pituri plant *(Duboisia Hopwoodii) *are used to stupefy the emu. The plan...is to make a decoction in some small waterhole at which the animal is accustomed to drink. After drinking the water the bird becomes stupefied, and easily falls a prey to the ...spear.

Roth [16] (in North-West Queensland) however rejected these claims and stated that 'pituri is certainly never used in any of these districts for contaminating the water-holes with the object of drugging the birds and animals drinking therein.'

Spencer and Gillen's work [36] confirmed that *N*. *suaveolens *was 'used after preparation, for chewing'. Their noted difference between the use of *Duboisia *and *Nicotiana *spp. would seem to be unambiguous except when Footnote 1 on page 611 [36] is scrutinized - it describes bags that 'are often used for carrying *pituri *in, and are similar to the well-known dilly bags of other tribes. *Pituri *consists of the dried leaves of *Duboisia Hopwoodii ***and is used as a narcotic by the natives**' (emphasis added). The Johnston and Cleland [42] essay on Central Australian Aboriginal populations begins to provide lucidity to the discussion on the identity of pituri:

Though the plant usually associated with the drug [pituri]...is mentioned as *Duboisia Hopwoodii*, the narcotic used for chewing in the greater part of Central Australia is not that species, but some kind of tobacco, such as *Nicotiana excelsior, N. Gossei *...

Hicks and LeMessurier [39] went further and explained that:

in the area north, north-west, and south-west of Alice Springs within a radius of 300 miles, [people] chewed, under the name of "pituri" the leaves of a least two varieties of Nicotiana [and] ...they wished to indicate that it [*D. hopwoodii*] was "pituri", but only used when *real *"pituri", i.e. Nicotiana, was unobtainable. At last it was disclosed the essential nature of the confusion as to the plant actually used for chewing.

Endeavouring to explain the variability in past chemical analysis of *D*. *hopwoodii*, Hicks supposed that, historically, plant matter of both genera may have been mixed together. Since the samples had to travel vast distances before laboratory analysis the 'friable Nicotiana would have been pulverised to an amorphous powder. The hard Duboisia fragments would still be physically identifiable. When steam-distilled with lime, understandably the mixture would have yielded nicotine' [9]. Eventually, Hicks and LeMessurier [39] established from specimens collected in South and Central Australia that it was not nicotine but d-nornicotine, a potent chemical four times as strong as nicotine that was the active and toxic principal in *D. hopwoodii *from that region. Bottomley and White [43] subsequently demonstrated that nicotine and nornicotine are usually both present. In an analysis of 67 *D. hopwoodii *samples from Western Australia collected from separate locations, and a variety of soils over a four month period, only four demonstrated a complete absence of nicotine, with all showing a wide variation in nornicotine (0.1 and 4.1%) and nicotine content (0 and 5.3 %). Further investigation established that the plants of Western Australia and Western Queensland contained mainly nicotine whilst those of South Australia and Central Australia contained nornicotine [23]. Barnard [23] and Watson, Luanratan and Griffin [44] asserted that due to the different regional soil, in particular salt content and pH, and with different seasons and rainfall, the *D. hopwoodii *produces differing levels of nicotine and nornicotine. Thus the different potency outcomes, from elation and rapture (those with high nicotine levels) to catalepsy and death (those with high nornicotine levels) explain the differing use of pituri throughout the Aboriginal tribes.

Aiston's [18] commentary substantiates Aboriginal chewers' understanding of ethnobotanical variability when he notes '...the pitcheri tree...grew in an area which extended from about due west of Bedourie, down to about opposite Birdsville, just over the Queensland border. Down to the south the trees were reckoned *kudna*, i.e. rotten, or no good'.

### Trade routes

Pituri (as both *D. hopwoodii *and *Nicotiana *spp.) held, and continues to hold, a position of importance and value in Aboriginal life, not only in terms of the powerful psychological and physically addictive effects of its nicotine content, but in terms of its role in social interaction and its dominance as a bartering commodity within and between tribal groups. There was a vast network of trade routes that linked Aboriginal groups in Australia [45]. Prized possessions were sought and bartered along these routes with 'pituri' consistently being cited as equivalent in status to boomerangs, spears, shields and ochre [15,16,20,42,46-49]. Given the misunderstandings of the term pituri, the presence across Australia and particularly the Central Australian region of both *D. hopwoodii *and over 20 species of *Nicotiana*, and the differing substances 'pituri' referred to, it is now not possible to ascertain if this 'pituri' was *D. hopwoodii, Nicotiana *spp., both, or something else that has now been lost with the passage of time. George Aiston [18] describes this very well when he says:

a great trouble to investigators is the lack of words in the aboriginal language; the one word pitcheri had to deal with the whole subject; the bush, *Acacia salicina*, in this country (Lake Eyre district) was more often known as pitcheri than by it's native name *wirra*. The ashes resulting from burning *wirra *bush tips were always known as pitcheri. So that any one asking would be shown perhaps half a dozen trees which would all be quite truly called pitcheri, although they only supplied supplementaries to the real substance.

### Ethnopharmacology - *Nicotiana *preparation and use

Today, *pituri *is one of several common terms used by both Aboriginal and Europeans in Central Australia to describe plant substances that are retained in the mouth for the purposes of nicotine extraction. In Central Australia chewing by Aborigines is common and restricted to wild *Nicotiana *spp., not *D. hopwoodii*. A range of *Nicotiana *species are reportedly used in the Central Australian region, however nicotine levels vary with species, environmental, and preparation factors - the preferred species are *N. rosulata *subsp. *Ingulba *(J.M.Black) P. Horton and *N. gossei *Domin [4,15,50]. In the context of the Australian Aboriginal ethnography, the chewing of the *Nicotiana *spp. mirrors the tobacco 'sucking' practices described by Wilbert [3] of several South American tribes. Pituri is prepared by breaking up fresh or sun/fire dried leaves into pieces, mixing with ash and chewing to form a 'quid'. A range of wood is burned to form the ash; some species mentioned in the literature include *Acacia *spp., *Grevillea *spp. and *Eucalyptus *spp.[15,18,51]. *Acacia salicina *is one of the plants most preferred for the ash, which Higgin [52] reported contained calcium sulphate at 51%, a 'much larger quantity than in any other ash at present known to us'.

The quid is held in the lower lip and buccal cavity or the cheek for extended periods of time. The oral cavity has a thin epithelium and rich blood supply, consequently the absorption of the nicotine is rapid and avoids first pass metabolism. Nicotine is an alkaloid so the addition of an alkalizing substance such as ash would be expected to raise the pH and therefore reduce its ionisation and increase lipophilicity, which would potentiate both the release of nicotine through the plant cell wall and the absorption through the mucosa of the mouth. The quid is passed from one chewer to another before the owner returns the quid to their own mouth. When not in the mouth, the quid is stored in the post-auricular space (behind the ear) under a breast, or under an arm-band or a head-band [15] - all are sites allowing for the continued absorption of nicotine via the transdermal route, which suggests similarity to the use of a commercial nicotine patch. Furthermore, a final quid is prepared and retained in the buccal cavity overnight, thus there is a potential that exposure and absorption of nicotine for chewers is continuous.

### Nicotine pharmacology and nicotine narcosis

Throughout the literature, and commencing with the very first notations of pituri use, is the continuous commentary that the chewed substances are 'narcotics' or are being chewed for their 'narcotic effect' [13-15,17,20,32,33,35,39,49,53]. The world of the late 1860s through to the 1940s had a vastly different usage, understanding, and convention around *narcotic *compared to contemporary practice. In 1882 [54] narcotics were defined as having the ability to:

...diminish the activity of the nervous system, produce sleep, and in most instances relieve pain, but which also are capable, if given in small repeated doses, of exciting the nervous system; by this they are distinguished from the class of medicines named *Sedatives*.

In 1892 'the drugs employed to produce sleep...were selected from the group of narcotics' [55]. By 1909 the definition of narcotic had expanded to 'any drug that produces sleep or stupor and at the same time relieves pain' [56]. Certainly the narcosis and other physiological effects noted by the early explorers and authors indicated that pituri chewing fitted these definitions and understandings.

While Bryant, Knights and Salerno [57] confirm that by definition the term *narcotic *literally means 'causing numbness, sleep or unconsciousness, and so could apply to all central nervous system depressants', the term *narcotic *in 2010 is generally connected with criminality and is applied more commonly to illicit drug use and the behaviours around that. The use of *narcotic *is therefore discouraged in a health context, and the term 'opioid' is now the preferred term [58]. The continued use of *narcotic *in reference to tobacco addiction can create confusion, particularly as tobacco self- administration is legal (for adults). The association of nicotine with narcosis is demonstrated by Benowitz [59]. Once in the bloodstream, nicotine crosses the blood-brain barrier and is rapidly distributed to the brain with an almost instantaneous effect on the central nervous system. The action of nicotine is complex and multifactorial - both Benowitz [60] and Grenhoff and Svensson [61] illustrate that the effect of nicotine is moderated by the amount of nicotine already in the body, the target organ, the prevalent autonomic tone and prior exposure history (tolerance), the time passed since the last exposure to nicotine, stress level and even the time of day.

Nicotine is a cholinergic drug and acts on nicotinic cholinergic receptors in the brain and other organs of the body; therefore it has the capacity to affect neurotransmission and consequently has the potential to alter conscious states, verifying Curl's [11] observation of the pituri users' trance-like state. Nicotine has a classic biphasic action dependent to some degree on the above variables. Initially nicotine acts as a stimulant, enhancing the release of neurotransmitters such as acetylcholine, norepinephrine, dopamine, beta-endorphin and serotonin - speeding up many body reactions; actions which sustain both the physical and psychological addiction to the substance and which would have produced the increased level of excitement required prior to tribal battles. Bryant, Knights and Salero [57] note that conversely after repeated doses, nicotine has depressant-like actions, slowing down reactions by inactivating cholinergic receptors directly, but indirectly, producing a wide range of physiological actions. This depressive action substantiates the 'narcotic' effects, or in the extreme, cataleptic effects, noted by the early authors and would have enabled such activities as the arduous treks without food or water that the Aboriginal people routinely undertook.

Seeking a state of altered consciousness through the use of nicotine is not confined to the Australian Aborigine. The ability of tobacco to achieve this commonality of addiction and reward exists despite the heterogeneity of the human population. For example, Wilbert's [3] work details tobacco smoke-induced trance states and hallucinations in traditional South American Indians which parallels T.S.Eliot's [62]*Portrait of a Lady *- dance, dance/Like a dancing bear,/Cry like a parrot, chatter like an ape/Let us take the air, in a tobacco trance'. The need for nicotine is so overwhelming, that, despite physical harm, addicts seek to gratify their cravings by its use. Tjakamara [63] describes the craving for *mingkulpa*, a Pintupi word used for all tobaccos and therefore translated to mean pituri:

Don't bring back the weak leaves - bring back the strong ones. Let us try it first. Don't bring back the weak leaves without trying it. Let us bring back ash tree to mix with the pitcheri. Let us eat it together with the ash, we who are starving for pitcheri. Let us eat it so it can burn our throats.

### Health outcomes - unanswered questions

Whilst pharmacological studies undertaken using commercially prepared smokeless tobacco demonstrate that chewers achieve substantial nicotine blood concentrations at least equivalent and often more than inhaled tobacco users (Table 2) [2,59] the level and extent of research examining the general health outcomes of smokeless tobacco use is inadequate compared to the health evidence that exists for inhaled tobacco use. The leading report into the health outcomes [2] and confirmed by the few studies in the field [64-68] identified that the general health outcomes for smokeless tobacco users 'are expected to be the same' as for inhaled cigarette users which includes addiction, hypertension, increased cardiac disease, increased stroke and increased rates of cancer including oral cancer. These outcomes are based on the evidence that it is not simply the inhalation of smoke that is harmful, the administration of nicotine *per se *is damaging. More recent work supports this hypothesis, with Shah [69] demonstrating albuminuria and abnormal renal function in tobacco chewers and Gupta et al.[70] indicating that chewers had systolic and diastolic blood pressures, resting heart rates, total cholesterol, LDL cholesterol and triglycerides comparable to smokers.

**Table 2:** Comparison of blood nicotine concentrations (ng/ml) following four different methods of administration

Administration method	Period of exposure (min)	Peak blood concentration ng/ml	Concentration (ng/ml) at	
			30 min	120 min

Cigarette smoking	12	15	10	6
Oral snuff	30	15	15	12
Chewing tobacco	30	14	14	12
Chewing nicotine gum (4 mg)	30	9	9	7

Data taken from Benowitz et al.[67]. Data represents average values for the same 10 subjects, each with previous nicotine exposure, prior to nicotine administration via differing methods.

## Conclusion

This review summarizes the scientific development in understanding the Australian Aboriginal ethnographical knowledge, habits and practices around *D. hopwoodii *and *Nicotiana *spp. Joseph Bancroft, whose pursuit of the true nature of *pituri *initiated vigorous pharmacological endeavour and grew an industry out of his persistence, considered that *D. hopwoodii *'should be known by the Aboriginal title' and 'propose[d] therefore, to name it *Duboisia Pituri' *[20] - despite his efforts, the '*hopwoodii' *remained.

There has been no research undertaken exploring the health outcomes of *pituri *for Australian Aboriginal populations. The recognition that pituri is a wild tobacco plant, and that there is at least a resemblance in the administration and absorption between pituri and commercial nicotine patches and gum allows researchers to draw upon the known health outcomes of commercial chewed tobacco. In the same way as commercial tobacco chewers self-regulate their dose of nicotine, wild tobacco chewers modulate their dose by varying the length of time a quid is held in the mouth, the frequency of quid changes and the amount of nicotine-rich saliva ingested or expectorated. In commercially prepared tobacco products the nicotine content is relatively constant and controlled through production methods, but these controls are clearly absent with the use of wild plants. Aside from the likely variable levels of nicotine within and between *Nicotiana *spp, the use of pituri by Australian Aborigines is markedly different due to the addition of ash and the continuous administration of nicotine either through oral or transdermal administration. Knowledge and awareness of the health implications of pituri use is an area for inquiry and research given the distinctions between commercially prepared smokeless tobacco and pituri.

## Competing interests

The authors declare that they have no competing interests.

## Authors' contributions

AMR conceptualized the theoretical framework, conducted the literature review and wrote the paper; KJS appraised the botanical information and contributed to that section; FB overviewed the framework of the paper and contributed to the structure and flow of the paper. All authors read and approved the final manuscript.

## Funding

No funding source.
